# A New 3D Object Pose Detection Method Using LIDAR Shape Set

**DOI:** 10.3390/s18030882

**Published:** 2018-03-16

**Authors:** Jung-Un Kim, Hang-Bong Kang

**Affiliations:** Department of Media Engineering, Catholic University of Korea, 43-1, Yeoggok 2-dong, Wonmmi-gu, Bucheon-si, Gyeonggi-do 14662, Korea; amysh@catholic.ac.kr

**Keywords:** multimodal sensor fusion, object detection, feature enhancement

## Abstract

In object detection systems for autonomous driving, LIDAR sensors provide very useful information. However, problems occur because the object representation is greatly distorted by changes in distance. To solve this problem, we propose a LIDAR shape set that reconstructs the shape surrounding the object more clearly by using the LIDAR point information projected on the object. The LIDAR shape set restores object shape edges from a bird’s eye view by filtering LIDAR points projected on a 2D pixel-based front view. In this study, we use this shape set for two purposes. The first is to supplement the shape set with a LIDAR Feature map, and the second is to divide the entire shape set according to the gradient of the depth and density to create a 2D and 3D bounding box proposal for each object. We present a multimodal fusion framework that classifies objects and restores the 3D pose of each object using enhanced feature maps and shape-based proposals. The network structure consists of a VGG -based object classifier that receives multiple inputs and a LIDAR-based Region Proposal Networks (RPN) that identifies object poses. It works in a very intuitive and efficient manner and can be extended to other classes other than vehicles. Our research has outperformed object classification accuracy (Average Precision, AP) and 3D pose restoration accuracy (3D bounding box recall rate) based on the latest studies conducted with KITTI data sets.

## 1. Introduction

Driver assistive technology to be mounted on a commercially available vehicle is rapidly developing. Although only a few years ago, it was equipped with level-0 technology that warns the driver through alarm sounds. Recently, sensors have been used to detect the lane through the ACC (active cruise control) as well as provide level-2 autonomous driving technology, such as LAKS (Lane Keeping Assist System), which supports steering. Some manufacturers [1], such as Tesla, also apply Level-3 technology, a partial autonomous navigation technology that includes automatic lane change. The development of computer vision technology played a key role as the background for this research. Vision technology analyzes information from various domains collected through CCD (Charge-Coupled Device) cameras, radar, ultrasonic sensors, etc., and makes it possible to judge the situation from a human perspective. In particular, deep learning-based research such as Alexnet [2] of the CNN (Convolutional Neural Networks) structure showed a dramatic improvement in accuracy and speed compared with a computer vision algorithm using an existing hand-crafted feature. These methods can be used in various fields. However, the autonomous driving field requires a high accuracy and computational speed because it needs to recognize surrounding objects in fast moving vehicles. In this respect, the object detection method based on deep learning was a suitable solution and in fact, many studies have been carried out. Most studies in this area use CCD images as input. CCD images are one of the most commonly used inputs because they are cheap and can provide information that is the most similar to human vision. However, it is difficult to acquire sophisticated 3D spatial information using a single CCD image alone, along with a significant calculation cost. Moreover, it is hard to analyze situations such as occlusion or truncation caused by objects. Even with the same object, there are disadvantages that many variables are generated depending on the light source and noise.

Laser sensors, such as LIDAR(Light + RADAR) [3], on the other hand, provide the freest sensor information for CCD image problems. Radar is a sensor that emits a laser in a radial manner, estimates the depth through reflected time, and projects each point in 3D space to obtain 3D spatial information about the vehicle. As a result, stereoscopic spatial information irrelevant of the light source can be acquired at the same time. Thanks to these advantages, many studies have been conducted to classify the objects around a vehicle using LIDAR information, but this has not become mainstream. The main reason is that the density is irregular due to the characteristics of LIDAR, which radiates a constant channel laser around the sensor and obtains information. The density of the point per unit area is 10 times or more different depending on the distance, and the LIDAR point is not evenly distributed to the target object, so that the same object is expressed completely differently. Two representative methods have been proposed to solve this problem.

First, vote3D [4] divides the space of all LIDAR points into a 3D voxel grid and quantizes them to the voxel level to learn the distribution of cells. However, because LIDAR is fundamentally less dense with distance, it cannot express long distance objects while maintaining structural consistency even if it is changed to a 3D voxel form. As a result, learning and classification through voxels is clearly limited and performance degrades compared to CCD image-based methods. Second, the MV3D [5] study projects LIDAR to the bird eye view and the camera view. In fact, the researchers proposed a method to compensate for the lack of information due to low density by mapping the height, density, and intensity information of each point. The 3D bounding box proposal is generated using LIDAR distribution information from a bird’s eye view and converted into a 2D Proposal to perform learning at two points. However, this method relies on LIDAR information in a bird’s eye view for both object proposal generation and 3D pose estimation. The researcher’s supplemented the feature by adding some information, but did not overcome the limitations of LIDAR’s object expressiveness that was weakened by distance. As a result, the objectivity score and direction of the 3D bounding box are less accurate.

**Figure 1:**
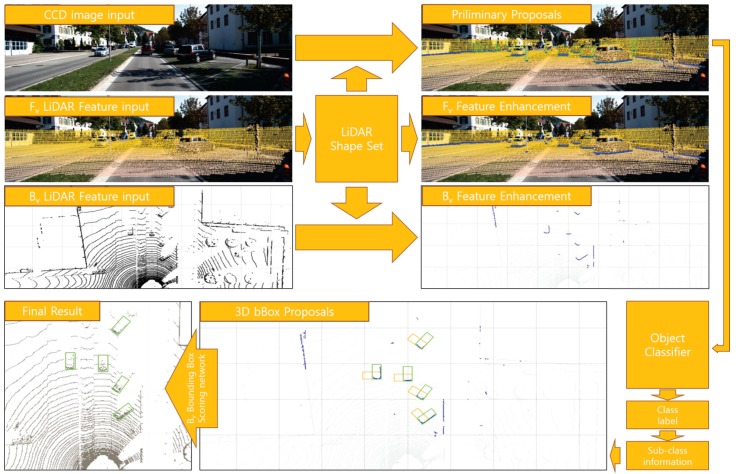
System overview. The proposed method performs 3D object classification and pose estimation using CCD image and LIDAR point cloud as input. Using the LIDAR Shape set created in the LIDAR Front view, we supplement the LIDAR Feature and create 2D and 3D bounding box proposals to help accurately detect objects in 2D and 3D.

Therefore, in order to effectively use LIDAR information, it is important to solve the problem of inaccurate representation of the object because the density of points per unit area is greatly reduced as distance increases, without impairing LIDAR’s ability to express 3D spatial information. In order to solve this problem, we propose a simple method to reinforce LIDAR point cloud object shape points and to use this proposal generation, 3D object classification and pose restoration. After separating the points that are likely to be objects in the LIDAR point cloud, we find the shape region of the object in the 2D front view. The shape of an object creates a shape set that describes the shape of an object based on depth and density gradients. This LIDAR shape set adds to the LIDAR feature maps to complement and classify the description of an object. Figure 1 outlines this entire process.

Given that the LIDAR Shape set represents the outline of an object in a 3D space, 2D and 3D proposals can be generated without adding a separate proposal generation method by adding the aspect ratio information per class.

This makes it possible to perform far fewer classifications than the conventional method of generating and classifying proposals for the entire image region, and to find objects with a higher accuracy with the same number of proposals. We demonstrate this through a comparative evaluation based on the KITTI dataset (http://www.cvlibs.net/datasets/kitti/) [6].

Our method consists of four steps. The first is the process of organizing the LIDAR Point to create the object shape and the LIDAR set, and the second is the process of creating a simple preliminary proposal using the LIDAR set. Third, we introduce a fusion classifier that classifies objects by concatenating the Front view CCD features and the Shape enhanced LIDAR features. Finally, the 3D pose of the object is determined by computing objectness scores of the extended bounding box based on the RPN (Region Proposal Networks) [7] network using the LIDAR feature of the bird’s eye view and the classification result of the preliminary proposal.

In summary, our contribution is as follows:

- We propose a LIDAR Shape set representing the shape of an object and compensate for the disadvantages of the LIDAR feature whose representation varies greatly with distance.
- We propose a LIDAR Shape set partitioning method that creates 2D/3D proposals based on intuitive object local information.
- We show a 3D object pose reconstruction method that combines subclass information with a feature level fusion classifier that combines CCD and LIDAR information. The proposed method was tested and verified in a public KITTI benchmark data set. The code will be submitted to the benchmark competition after optimization.

## 2. Related Work

Before explaining the proposed methodology, we briefly review existing methods in the field of proposal generation, object detection and multimodal fusion.

**2D Object Proposal Generation** Classifiers such as the initial RCNN [8] and Fast RCNN [9] can separately classify objects using the CNN Feature of the corresponding region by determining the area where the object can exist using a conventional method of proposal generation Respectively. These traditional proposal creation methods are called selective search [10] and Edge Boxes [11]. A selective search creates a 2D bounding box by predicting the area in which objects can exist through the segmentation boundaries of the 2D image. EdgeBoxes uses the shape of the object as a closed edge shape and groups each pixel to calculate the similarity of the boundaries. Both of these studies showed good results, but both the proposal generator and the CNN classifier are performed independently. Therefore, it is inefficient due to redundant operation in the feature generation part for judging the object. For this reason, Faster RCNN researchers suggested RPN. They determined that the ConvNet feature of the CNN architecture represented the object well. Faster RCNN increases the efficiency of the process by generating a proposal via RPN and then sharing the convnet feature from the proposal generation process with a later associated RCNN classifier.

**3D Object Proposal Generation** Like the 2D object proposal, the 3D proposal generation method creates a small set of 3D candidate boxes to detect most objects in a 3D space. To do this, 3DOP [12] designed a depth feature map in the 3D point cloud to evaluate the 3D bounding box set. Another case, Mono3D [13], uses some subdivision functions to generate a 3D proposal from a single image using a ground truth label. Both 3DOP and Mono3D use a hand craft feature set to estimate the bounding box. Deep Sliding Shape [14] uses more powerful in-depth learning capabilities. However, this method uses a 3D voxel grid. In CNN, the 3D input is processed using a 3D convolution, so the overall calculation process is complex and slow. To solve this problem, we create 2D and 3D proposals as a way to filter the object information of a projected LIDAR Point cloud in 2D space. The shape set of the objects created in this process is shared to enhance the LIDAR information used for object classification.

**Object detection in 2D** A few years ago, alexnet led the way to CNN-based object detectors such as Over-Feat [15] and R-CNN, which greatly improved the accuracy of object detection. OverFeat adopts ConvNet [2] as the sliding window detector of the image pyramid and adopts a strategy similar to the initial neural network detector. R-CNN was developed as a region-based detector that generates a conv feature map for each proposal generated through a proposal generator, such as Edgeboxes or selective search, and optimizes the network. SPPnet [16] has shown that these region based detectors can be applied more efficiently when using feature maps extracted from a single scale image at once. Recent object detection methods, such as Fast R-CNN and Faster R-CNN, use a feature map calculated from a single-level Fully Connected (FC) layer to provide a balance between accuracy and speed. However, there is the disadvantage that it is difficult to classify small objects accurately because the feature information that can be used for detection is too small. To overcome these drawbacks, several methods have been devised that use convolution feature maps generated by pooling at multiple scales. A recent approach using these multiple layers improves detection and segmentation performance by using multiple layers created within CNN when detecting objects. The Fully Convolutional Network (FCN) [17] sums the subscores of each category for multiple classes to compute the semantic segmentation region. Other methods, such as HyperNet [18] and SDP [19], combine multi-layered convolutional feature maps together or entail the use of an appropriate level of convolutional features depending on the size of the Region of Interest (ROI). Such a method can be used to obtain feature information suitable to the size of the object. YOLO [20] and SSD [21] used a multi-scale feature detection method at the grid cell level by dividing the entire area into grid units.

**Object detection in 3D** Most of the existing methods use 3D voxel grid representations to encode 3D point clouds. The sliding shape [14] and Vote3D [4] classify 3D voxel structures encoded with geometric features using an SVM [22] classifier. Some of the recently proposed methods use 3D convolution networks, which require costly calculations. In the case of VeloFCN [23], each point in a point cloud is projected onto a front view taken with a CCD camera, without using a 3D voxel, to obtain a 2D point image. Researchers using this method learned 3D ground truth bounding boxes on 2D point images and convolution feature maps using FCN networks. In addition, a method of detecting 3D objects using only images without using LIDAR has been studied. 3DVP [24] consists of a 3D voxel pattern defined using a CAD model and ACF detector [25] set to perform 2D detection and 3D pose estimation. 3DOP uses an energy minimization approach to reconstruct the space in the stereo image to produce a 3D bounding box proposal, which is provided as an R-CNN pipeline for object recognition. Mono3D has a similar structure to 3DOP, but uses a single image to create a 3D bounding box. In addition, some studies have used a timeline sequence composed of multiple frames to combine the motion and ground estimation of the image to create a 3D bounding box.

**Multimodal Fusion** The KITTI dataset provides a large amount of data that can be obtained from a vehicle such as: a CCD image, stereo image, LIDAR point cloud, and GPS information. It distributes the calibration matrix and sample code between each data point. It also provides a ground truth label for the object learning of each piece of data. However, most studies on object detection use only the information from one sensor and there are not many studies that use the information from multiple sensors together. Mao et al. [26] used a combination of color image, depth map, and optical flow to detect pedestrians within a CCD image. Mao analyzed the effects of various types of features on detecting pedestrians through experiments and studied the optimal combination of features and methods. MV3D [5] is the most similar to our study as it represents a way of generating a LIDAR-based proposal by mapping all of the points in the LIDAR point cloud to the pixel planes of a bird’s eye view. Then, they extended the feature map to three points by adding height, density, and brightness information to each point. Extended feature maps are used for RPN learning and generate 3D bounding box proposals. Next, the generated proposal is used as ROI for object classification in a bird’s eye view and front view. The bird’s eye view uses the LIDAR Feature used in the RPN similar to Faster R-CNN, and the front view uses the CCD and LIDAR Feature as input to output class labels and bounding box regression results for an object. Finally, the final 3D bounding box is classified and assigned a class using the three results deep fusion method.

However, this method creates object proposals and features using a sparse LIDAR point cloud. Given that the LIDAR Feature, which changes its shape according to the distance, is used for learning and classification as is, it is difficult to classify an object at a long distance and the learning efficiency is deteriorated.

We have devised a way of supplementing the object’s boundary shape in order to better represent LIDAR-composed objects. This shape set is added to the existing LIDAR feature to help stabilize object representation. We also split the shape into object units, create a simple 3D proposal, and estimate the class and 3D pose of the object using two views and a fusion method consisting of three features.

The following chapters introduce the specific structure and process of this method.

## 3. Overview

**Figure 2:**
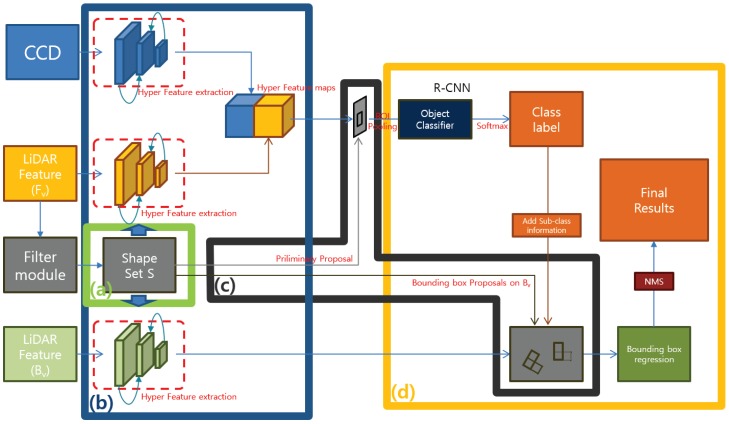
Network architecture overview. This figure shows the structure of the proposed method to object detection and 3D pose restoration using 3 input features.

Our proposed method consists of four modules. ‘LIDAR Feature map complementation’, ‘LIDAR Shape set generation’, ‘Preliminary Proposal Generation’, and ‘3D Pose restoration’. Figure 2 shows the flow of this process structurally. The LIDAR Feature map complementation module (Figure 2a) describes a complementary method of the obscure LIDAR feature map used to represent objects clearly. Through simple 3D voxel grid filtering, the ground is removed, and object shape points are reinforced to obtain clear boundary shape information. We can obtain a large amount of information from the boundary shape of the object, such as the corner position of the object, the shape of the object, and the occlusion relationship with neighboring objects. For example, adding shape information to a uniformly distributed existing LIDAR feature makes it possible to more clearly represent the object, and the orientation of the object can be estimated using the direction of the shape edge connected to the corner position.

The LIDAR Shape set generation module (Figure 2b) divides the LIDAR shape set into object units using a depth gradient and density gradient, and generates preliminary proposals. The proposal generated by the partitioned shape set creates boxes only for the visible region of the image, and serves to separate only the classes of each set without estimating the entire area of an object. If the classified class is a rigid body model, a 3D bounding box is created by adding the previously defined sub category information in the fourth step.

The Preliminary Proposal Generation module (Figure 2c) is a feature fusion network for classifying objects using preliminary proposals. Our suggested method is similar to MV3D, object detection and classification, and 3D pose restoration are performed using two front view inputs and one bird’s eye view input. However, since we generate the LIDAR-based proposal limited to the visible object area in the proposal creation stage, each ROI includes both the CCD feature and the reinforced LIDAR feature. Therefore, we concatenate at the feature level to perform object classification based on richer features. In the case of the bird’s eye view, fusion is not possible at the feature level because it differs from the front view, but it is used as a role to determine the pose of the object by learning in RPN form.

The 3D Pose restoration module (Figure 2d) is to determine the 3D bounding box using the 3D information of divided shape sets, classified class labels and sub information. A rigid body type class can determine the average width, height, and length of the class in advance and extend the 3D bounding box in the edge direction connected to the corner coordinates of the LIDAR Shape set. However, if the length of the edge is not sufficient, there are two bounding boxes that can extend from one corner. We solved this problem by computing objectness score with the object proposal generated through the LIDAR feature of the bird’s eye view.

## 4. Method

This chapter explains how to handle the LIDAR Point cloud to improve the appearance of the LIDAR feature used for object detection and classification. We also describe the process of creating 2D/3D proposals using a LIDAR Shape, classifying objects via a fusion classifier, and restoring 3D pose.

### 4.1. LIDAR Feature Map Complementation

**Object Seperation** We reinforce the LIDAR Point cloud L along the outermost border of an object to stabilize the shape of the irregular object that the LIDAR represents. Given that the reinforcement work is limited to the object, l, belonging to the entire L should be differentiated into ‘object’ and ‘ground’. This differentiation is done in a very simple way. We integrate the entire point cloud into a 3D voxel grid. This 3D grid is a data space that divides the entire three-dimensional space of the *x*-*y*-*z* axis by 20 cm × 20 cm × 10 cm. Each cell appears in one of three states. If the number of points is greater than a certain threshold value, $C^{oc}$ (‘occupied cell’), $C^{ob}$ (‘object cell’), then subdivide a cell with a threshold below $C^{gr}$ (‘ground cell’). We can grasp the distribution and shape of the points in all cells of the 3D grid through this process.

In order to enhance the object expressiveness of the $C^{ob}$ that describes the ‘object’, we created a shape set *S* that represents the shape edges of the object by projecting the $p^{3D}$ belonging to the $C^{ob}$ to the 2D space.

Filtering the LIDAR Point cloud in 2D space has several advantages over direct filtering of the point cloud in the 3D view. Due to the discontinuous pixel space characteristics, the filtering process such as noise reduction is simplified, and the directivity of both features is guaranteed when LIDAR and CCD are fused. In addition, since the multi-view fusion problem between CCD and LIDAR classifies the objects in the camera front view ($F_{v}$), it can also reduce the total amount of computation by ignoring LIDAR points that are not needed for the operation. For this reason, we create a LIDAR shape set *S* complementing $C^{oc}$ in a 2D space mapped 1:1 with an original 3D space.

**Figure 3:**
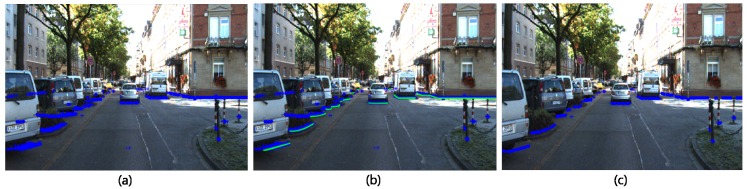
(**a**) A set of points $p^{2D}$ that project LIDAR Point $p^{3D}$ at the same height in a 3D front view; (**b**) The bottom edge of $p^{2D}$ projected on the front view(green highlight) coincides with the curve that represents the shape of the boundary of the object; (**c**) LIDAR shape set generated by the proposed method.

**LIDAR Shape set generation** First, we map all points $p_{i}^{3D}$ in $C^{ob}$ to the horizontal plane of the *x*-*y* axis with the same height and project it onto a CCD camera image as shown in Figure 3a. Given $p_{i}^{3D}$ = $\left\{x,y,z\right\}$ for all *i*, point $p_{i}^{2D}=\left(c,r\right)$ is calculated using Equation (1) below.

(1) $$p_{i}^{2D}\left(c,r\right)=\left\{\begin{array}{c} c=\left\lfloor atan2(y,x)/\Delta \Theta )\right\rfloor \\ r=\left\lfloor atan2(z,\sqrt{x^{2}+y^{2}}/\Delta \phi )\right\rfloor \end{array}\right.,\;\forall p_{i}^{3D}\left\{x,y,50\right\}\in C^{ob}$$

where *r* is the *x* coordinate of $p_{i}^{2D}$ projected on $F_{v}$, and *c* is the *y* coordinate. In the process of projecting $p_{i}^{3D}$ on one plane to extract the shape of the object, the reason why $p_{i}^{3D}$’s *z*-axis size is 50 is because the height that can universally describe the object near the vehicle on $F_{v}$ is 50 cm.

Then, using Equation (2), the points located out of the $F_{v}$ range among the points of the entire $C^{ob}$ are removed.

(2) $$p_{i}^{2D}\left(c,r\right)=\left\{\begin{array}{cc} p_{i}^{2D}\left(c,r\right) & if\;1<c<I_{w}\;\&\;1<r<l_{h}\\ REM(p_{i}^{2D}\;\&\;p_{i}^{3D}) & otherwise \end{array}\right.$$

where REM () is the remove function. *REM* does not delete $p^{2D}$ and $p^{3D}$ but excludes it from $C^{ob}$. Given that $p^{2D}$ is the projection of $p^{3D}$ with a fixed z-dimension in 3D space, $F_{v}$ is expressed as a scrambled shape rather than a general object shape (Figure 3a). However, if we connect the lowest point along each column of each image, we can see that it has an object boundary shape in $B_{v}$ (Figure 3b). Thus, if we remove all points leaving only the bottom pixel of each *x* axis column *j* of $F_{v}$, we can convert it to a LIDAR shape set *S* close to the object’s $B_{v}$ boundary shape. The formula is as follows:

(3) $$S=\arg\min_{c}\left\{p_{i}^{2D}\left(c,r\right)|round\left(r\right)=j)\right\},\;\forall j$$

where *S* is the set of minimum height points in the *j*th column in $F_{v}$. Figure 3c shows the result *S* of the curve deformation. Let $p_{k}^{2D}\in S$ be a set of boundary shape points. In some cases, noise points are left due to irregular reflection or distortion. We use Equation (4) to remove noise and perform point interpolation on the blank pixels.

(4) $$\begin{array}{c} S=G(M\left(s_{k}\right)),\;\forall k \end{array}$$

where *G* is 5×1 gaussian filter and *M* is the 3×1 median filter. If the movement of $p_{k}^{2D}$ occurs by *M*, $p_{k}^{3D}$ corresponding to $p_{k}^{2D}$ must also be moved. However, if we move $p_{k}^{3D}$ of $B_{v}$ by median filter to the *x*-axis, the shape differs greatly from what we see on $f_{v}$. Therefore, $p_{k}^{3D}$ of $B_{v}$ resets the coordinates using linear interpolation between $p_{k-1}^{3D}$ and $p_{k+1}^{3D}$.

**Figure 4:**
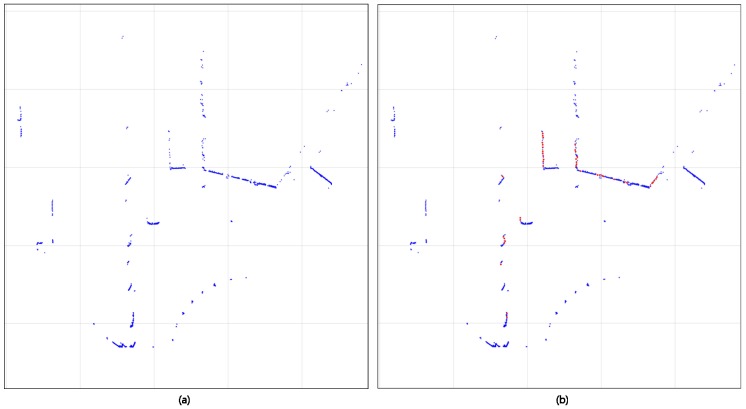
(**a**) Shape representation of an object. The long border edges of *y*-axis are relatively spaced from each other; (**b**) New points (red points) that reinforce the LIDAR shape set with a simple interpolation method.

During this process, we can classify the points in the shape region of an object by associating $p_{i}^{2D}$ and $p_{i}^{3D}$, which are 1:1 and connected at two viewpoints, with a minimum height of $F_{v}$. Although this method can easily present the shape representation of an object, the long edges of the *y*-axis are relatively spaced from each other, as shown in Figure 4a. To compensate, the following Equation (5) is used to add n points to the LIDAR shape set according to the *y*-axis distance of the neighboring points. Figure 4b is the resulting image of the completed LIDAR shape. This set of shapes is used to create 2D object proposals and enhance the LIDAR feature for classifying objects in addition to existing LIDAR Point clouds.

(5) $$\begin{array}{c} IP_{k}=floor\left(D/threshold\right),\\ D=\sqrt{{\left(p_{k-1}^{3D}\left(x\right)-p_{k}^{3D}\left(x\right)\right)}^{2}+{\left(p_{k-1}^{3D}\left(y\right)-p_{k}^{3D}\left(y\right)\right)}^{2}} \end{array}$$

In Equation (5) , $IP_{k}$ represents the number of points to perform a linear interpolation when the distance between two points exceeds the threshold. Given that the size of $C^{ob}$ is 20 cm, we used the threshold of 0.2 m (20 cm) in our research.

### 4.2. Preliminary Proposal Generation

**Figure 5:**
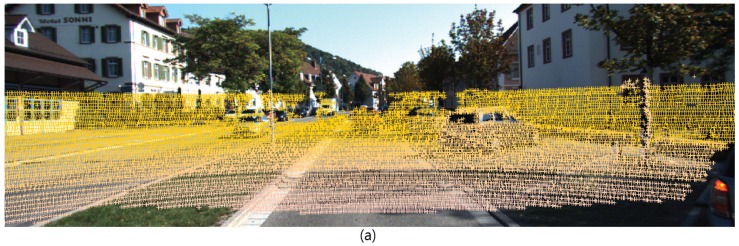

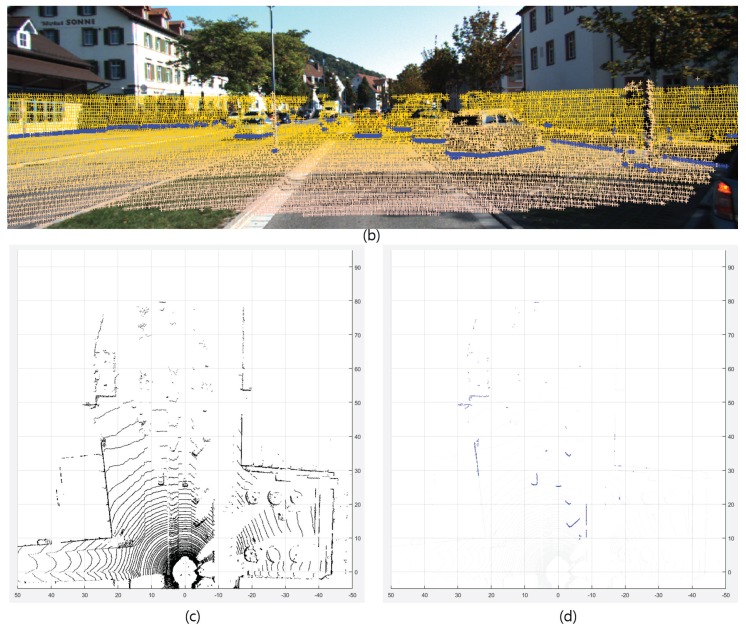
(**a**) The point distribution of the original $F_{v}$ LIDAR feature; (**b**) Points of $F_{v}$ LIDAR Feature added with LIDAR Shape set; (**c**) point distribution of original $B_{v}$ LIDAR Feature; (**d**) LIDAR points of $B_{v}$ with LIDAR Shape set added.

In the R-CNN classifier, the region proposal reduces the unnecessary classification process by summarizing the areas where the object may exist. Therefore, if the proposal is created using S as the shape for the object, it can be expected to have a higher classification rate compared to the number of proposals because it is built around the actual area of an object. *S* is projected in $F_{v}$, filtered and expressed in curves across the image. It is necessary to process the information in order to divide the curve data $p_{i}^{2D}=S$ into object units. We transform the 2D vector by mapping the density and depth of each point, transforming it into a 1D vector $b_{i}$, while ignoring the *c* value of all $p_{i}^{2D}$ that are spread in 2D space. The density uses the density of the $C^{ob}$ to which each $p_{i}^{2D}$ and matched $p_{i}^{3D}$ belongs, and the depth *d* can be expressed as the distance of each point from the vehicle, such as defined in Equation (6):

(6) $$\begin{array}{c} B=\left[b^{depth}\;;b^{density}\right]\\ \begin{array}{c} b_{i}^{depth}=\sqrt{p_{i}^{3D}{\left(x\right)}^{2}+p_{i}^{3D}{\left(y\right)}^{2}}\\ b_{i}^{density}=sizeof\left(C_{i}^{ob}\right),\;if\left(p_{i}^{3D}\in C_{i}^{ob}\right) \end{array},\;\forall i \end{array}$$

where *B* is a 2D vector set in which the depth and density of each point are linearly mapped to the horizontal position of shape *S* of $F_{v}$. We obtained a gradient for the two rows of *B* and subdivided the $s_{i}\subset S$ based on the points where the changes match. Figure 5 shows the intensity of each data mapped to *S* and the gradient strength of the splitting point. Given that $s_{i}$ is a group belonging to *S*, all $p_{i}^{2D}$ of $s_{i}$ have a 50 cm height based on the photographed vehicle. However, the actual height of the object may be different due to the bending of the ground. Therefore, it is necessary to reset the height of $s_{i}$ to the actual object position. The *y*-axis position of subset $s_{i}$ can be restored by the following Equation (7) :

(7) $$\begin{array}{c} p_{i}^{2D}=p_{i}^{2D}\left(c,r-dist\right)\\ dist=min\left(p_{i}^{2D}\left(r\right)\right)-\underset{z}{min}\left(C_{i}^{ob}\right) \end{array},\;\;\forall p_{i}^{2D}\in s_{i}$$

We obtained the *x* position, the width, and the bottom position of the *y*-axis of the group that are likely to be objects through $s_{i}$ using the method of adding the average height of classes. The typical height of a ‘car’ in the driving environment is 1.6 m, and in case of a ‘pedestrian’, the average height of 1.6 m is used as the standard. We can obtain all of the preliminary proposal bounding boxes $pp_{i}=\left\{x,y,w,h\right\}\in PP$ for $\forall s_{i}$. Then, extending the configured bounding box through three aspect ratios and three scales determines the final preliminary proposal PP.

We use LIDAR features and CCD images together, similar to multi-view, to classify objects into two viewpoints. Specifically, we use LIDAR features: $L^{B_{v}}$ in $B_{v}$, $C^{F_{v}}$ in a CCD image in $F_{v}$, and $L^{F_{v}}$ in the LIDAR feature. The most distinguishing feature from the existing research is that $L^{F_{v}}$ and ${}^{B_{v}}$ are complemented by using Shape set *S*, and proposal is generated using $s_{i}$ and 3D bounding box is constructed. $L^{B_{v}}$ is used for the scoring of the 3D bounding box created at this time. Figure 5a,c shows the existing $L^{F_{v}}$ and $L^{B_{v}}$, and Figure 5b,d shows the $L^{F_{v}}$ and $L^{B_{v}}$ with $s_{i}$ added.

### 4.3. Feature Fusion Network

Our proposed network consists of two parts. The first is an object classifier that concatenates two features of $F_{v}$ at the feature level. The second is a regression model of $B_{v}$ that determines the 3D pose of the object by verifying the 3D bounding box proposal created using $s_{i}\in S$.

**Figure 6:**
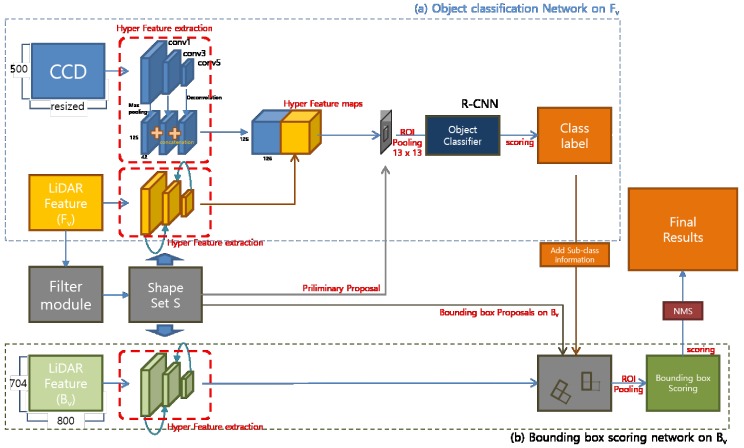
Network architecture detail.

As shown in Figure 6, we classify objects using CCD images and LIDAR images mapped to $F_{v}$. The latest 2D object detector uses RPN to generate an object proposal. However, we have created a preliminary proposal (PP) using a LIDAR shape set, *S*, in advance. The generated PP is created using the LIDAR point set $C^{ob}$, which is likely to be an object, so that it can achieve the maximum efficiency with a small number of proposals.

The existing proposal also learns the area including the foreground object if occlusion exists in the learning process. Therefore, there is a problem that is affected by the shape of the foreground object, regardless of the actual object. However, our proposed method does not require the consideration of occlusion in the learning process because it creates a proposal and classifies the object only for the area where the LIDAR point is projected, that is, the visible area.

The input features are CCD image and a LIDAR feature map projected on $F_{v}$. CCD Feature inputs uses three channels of $\left\{R,G,B\right\}$, and LIDAR Feature inputs maps depth, density, and intensity information to each point location. We also applied the feature supplement method of hyper features to classify small objects.

Both the CCD and LIDAR networks are pooled in a Conv1 layer and Deconvoluted in Conv5 layer to construct a hypernet that uses all three levels of features. However, we decided to concatenate the features of conv1, conv3, and conv5 generated by CCD and LIDAR, respectively, at the level unit and then to make them hyper features. Because the hyper feature map is a structure in which three levels of conv features are merged serially, it is more structurally natural to maintain the scale order for both inputs.

Given that we created the preliminary proposal in the previous step, it is easy to end-to-end train our network architecture. Proposal bounding box regression is performed in the next step using the LIDAR and subclass information of $B_{v}$. Thus, our loss function is defined on each RoI as the cross-entropy loss:

(8) $$\begin{array}{c} L=L_{cls}\\ L_{cls}\left(s_{c*}\right)=-log\left(c*\right) \end{array}$$

where $L_{cls}$ is classification loss, *s* is a softmax loss for class, and c∗ is the ground truth label for ROI.

### 4.4. 3D Pose Restoration

Figure 6a is a fusion classifier that classifies the objects in the region using PP made of $s_{i}\in S$. This classifier determines the class of PP and does not perform bounding box regression. $F_{v}$ features have information that is valid for determining the object at driver’s view point, Information is somewhat unstable to process 3D spatial information.

However, by using $B_{v}$’s LIDAR Feature, it is possible to intuitively express the 3D position and area of an object, and estimate the 3D orientation of the object from the shape of the $s_{i}$. Once the class label for the preliminary proposal is determined, the object’s 3D bounding box can be easily constructed by adding the general spectral ratio and minimum scale information of the class.

**Figure 7:**
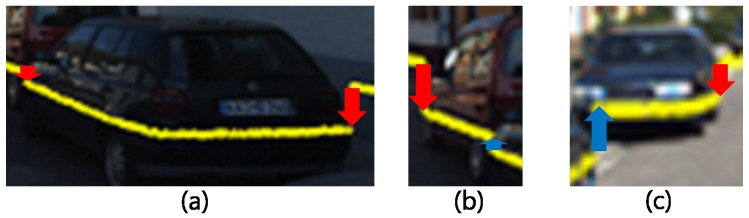
Occlusion states. (**a**) is a state in which both edges of $s_{i}$ are visible; (**b**) is a state in which the right side is obscured; and (**c**) is a state in which the left side is obscured. In our study, the height of $\forall s_{i}$ is readjusted to the height of the ground plane, but in this figure, the height of $s_{i}$ is adjusted to show the shape of object.

As shown in Figure 6b, the full 3D bounding box of the object is calculated using the objectness score of oriented bounding box generated by $s_{i}\in S$ with RPN using $B_{v}$’s LIDAR feature. $s_{i}\in S$ used in the generation of the PP has a neighbor relation to ∀i in the *x*-axis direction, given that the *z*-axis position of the camera expressing $F_{v}$ is located at the top of the vehicle. Therefore, the size of the *y*-axis of $s_{i}$ becomes smaller as the object closes in, and the neighboring $s_{i}$ and $s_{i-1}$ can grasp the occlusion state according to the relative y-value of the point on the boundary line. Figure 7 shows the three occlusion states that can be observed in the vehicle.

We use a point *P* that is not hidden according to the occlusion state as a reference point and create a 3D bounding box using the orientation of the edge connected to the reference point $P_{r}$. If there are two edges, then the edge *E* that has many points belonging to an edge is selected. Then, three points that are farthest from $P_{r}$ are selected and connected to the points with the medium *y* value to determine the reference angular $\theta_{r}$.

Given a $\theta_{r}$ and a $P_{r}$, we can create a 3D bounding box $B^{3D}$. Given that $s_{i}$ is the set of points closest to the sensor among the LIDAR points generated on the surface of the object, the maximum number of $B^{3D}$ using $E_{i}\subset s_{i}$ as the edge is two. However, $E_{i}$ is likely to be obscured by other objects and there may not be enough LIDAR points to represent an entire object. That is, it is difficult to determine the direction of an object by only the length of $E_{i}$. Therefore, we determine the final direction by assigning objectness scores for the two $B_{j}^{3D}$ regions in $B_{v}$. To obtain the objectivity score of $B_{v}$, the input of $B_{v}$ uses an LIDAR feature map $L_{B_{v}}$ composed of height, density and depth. $L_{B_{v}}$, like $F_{b_{v}}$, is reinforced by *S* for the shape feature.

**Figure 8:**
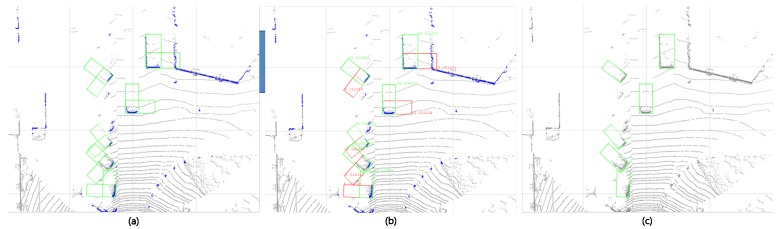
3D pose restoration process. (**a**) shows that two 3D border box suggestions are generated for each $s_{i}$; (**b**) shows that each bounding box is evaluated through a 3D bounding box scoring network; (**c**) shows that the bounding box with the highest score in (**b**) is selected as the final 3D box. In the extension method, multiple bounding boxes with intervals of 6 degrees are each scored and the final bounding box is determined by the NMS method.

If one of the two $B_{j}^{3D}$ of $E_{i}$ is selected through the softmax score, the final bounding box can be drawn on $F_{v}$ and $B_{v}$. Given that $s_{i}$ has the height information of the ground, a 3D bounding box is created which considers the *x*, *y* position as well as the *z* position. Figure 8 shows the final detection result.

## 5. Experiments

We create only two $B^{3D}$s per shape edge $E_{i}$ as a proposal. Therefore, the number of proposals used for classification is extremely small compared to existing methods. The average proposal generation number of 6536 images used in our test is 41.8. Ours-B of the graph is the result of the basic method of generating 2 proposals per $E_{i}$, ours-E is the expansion method of transforming theta at 6 degree intervals to increase the bounding box and then determine the box via $B_{v}$ for NMS results.

### 5.1. Implementation Details

Our suggestion was developed in a system environment consisting of an Intel i7-6700 CPU, NVIDIA titan X GPU with 12 GB memory, and 32 GB main memory. The software environment consists of Python 3.61, tensorflow-gpu 1.2, CUDA 8.0, and cuDNN 6.0 on Windows 10.

We used 6000 of 7481 images of ‘2D object’ categories provided by KITTI for training and 1481 of them for testing. In addition, 11,000 of the 16,055 data in the ‘tracking’ category were used for training and 5055 were used for testing.

The input image of the $F_{v}$ retained the original aspect ratio and the short side was fixed to 500. As a result, the CCD image of KITTI was up-scaled. In the case of $B_{v}$, it is discretized into a grid of 0.1 m units and the image size is 704 × 800.

The class category consists of ‘car’, ‘pedestrian’, ‘cyclist’ and ‘background’, and only objects with the ‘car’ label have a 3D bounding box with an aspect ratio.

### 5.2. Network Architecture

Our network is similar to MV3D with 3-view configurations using LIDAR and CCD, but there are many differences.

- The proposed network is divided into an object classification network and a 3D pose restoration network.
- The object classification network is based on a hypernet structure that integrates the features of conv1, conv3, and conv5 to support multi-resolution ROI, using LIDAR and CCD images projected on the front view.
- Unlike MV3D, the feature level fusion method is used. MV3D was also tested with an early fusion method, but because of its high dependence on $B_{v}$, the accuracy of $F_{v}$ fusion only declined. However, the proposed method improves the accuracy by processing the 3D localization and detection part separately from the B3D set as well as the reinforcing shape information of the $F_{v}$ LIDAR feature.
- The two hyper feature maps generated from LIDAR and CCD inputs are concatenated in series to become fusion feature maps.
- The fusion feature maps are ROI pooled to 13 × 13 in size and connected to the convolution layer for classification. Unlike hyperne or MV3D, the regression for the bounding box is performed separately by $s_{i}$, so only class label classification is performed.
- The bounding box regression is performed in the 3D pose restoration network.
- The 3D Pose restoration network is a LIDAR feature-based RPN in that the $B_{v}$ that evaluates the objectivity score for $B^{3D}$ is generated by *S* and determines the exact 3D pose for each $s_{i}$.

### 5.3. Training

The object classification network first pre-trains the hypernet structure using $F_{v}$ CCD and LIDAR as inputs. After the fusion feature maps are generated through the convolution layer of the two networks, four classes are learned using the ground truth ROI limited to the visible region. The network trains with SGD (Stochastic Gradient Descent) at a learning rate of 0.001 for 100 K iterations. We then lower the learning rate to 0.0001 and train 20 K iterations.

The 3D pose restoration network is an RPN structure using a 16 layer VGG network. We can train $B_{v}$’s LIDAR Feature maps regardless of class. We use $\left\{x,y,w,h,B_{obj}\right\}$ of the non-rotated full bounding boxes.

### 5.4. Metrics

We evaluate the 3D object proposal using a 3D box recall that computes the IoU (Intersection over Union) overlap of the two cubes, just like MV3D. The thresholds of 3D IoU were set to 0.25 and 0.5, respectively. 3D localization and detection accuracy were also evaluated. $AP_{loc}$ was calculated by calculating the average precision of the $B_{v}$ boxes projected on the ground plane. According to the agreement of KITTI, 2D average precision evaluates the 3D bounding box projected in a 2D space using an IoU threshold of 0.7.

### 5.5. Baselines

Given that this study performs object detection in 3D space, we also compare the LIDAR-based methods VeloFCN, Vote3D, and MV3D as well as CCD-based methods such as Faster R-CNN, 3DOP and Mono3D. For a fair comparison, 2D AP and 3D bounding box recall rate perform comparative assessments to provide results in the KITTI validation set.

### 5.6. Object Classification

We use average precision to verify the performance of 2D object detection. Therefore, we compared the classification rates for the three categories with the latest studies. The IOU threshold of the final 2D bounding box determined according to the KITTI evaluation method was set to 0.7, and the average precision for easy, moderate and hard was evaluated. Given that MV3D classifies only the car category, it is excluded from the evaluation of pedestrians and cyclists. Table 1 below show the classification accuracy for each class. Our method demonstrated an average AP of 94.9% for easy difficulty objects in the ‘car’ category. In addition, 80.4% AP was achieved in the hard case. This result is not only in comparison with MV3D, but also in comparison with the latest CCD-based methods which are advantageous for object classification. Table 2 compares the effect of shape set *S* on the LIDAR feature maps used in our two networks. In object classification, When LiDAR Feature is concatenated to CCD without Shape reinforcement, LiDAR Feature may degrade CCD classification performance. However, most of cases show improvement in performance when complementary shape of object is added. In this table, ‘C’ means CCD, ‘L’ means LIDAR features, and ‘S’ means LIDAR shape set.

### 5.7. 3D Pose Restoration

The 3D pose of objects is determined during the process of 3D proposals generated through the NMS process in $B_{v}$. However, in the case of MV3D, the *z*-axis position of the object is unstable according to the LIDAR point distribution due to a limitation of the information of $B_{v}$. However, our method repositioned the 2D and 3D bounding boxes using the bottom position of the 3D grid cell to which each object’s shape set $s_{i}$ belongs. We also added a translational anchor box of ±15% height considering the case that a LIDAR Point is not generated sufficiently. The proposed method can restore a more accurate 3D pose than the previous studies by leveraging these two processes. Table 3 shows the mean IoU rate between the 3D bounding box and ground truth detected in the car category of KITTI. Our method compensates the *z*-axis when generating each proposal, and creates a bounding box based on the straight line connected to the corner of the object, thus guaranteeing a high overlap rate.

### 5.8. 3D Proposal Recall

**Figure 9:**
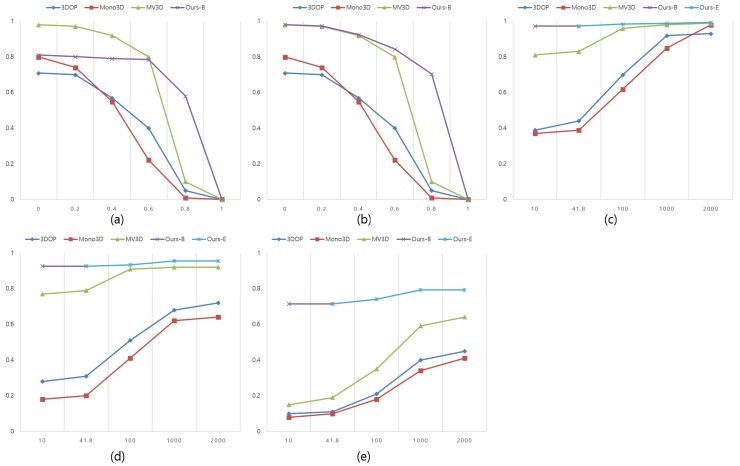
3D Proposal Recall change. (**a**) is a recall change graph for the whole ground truth of the KITTI; and (**b**–**e**) are results of applying the ground truth except for the object completely hidden in the foreground object as seen from the general driver’s viewpoint.

**Figure 10:**
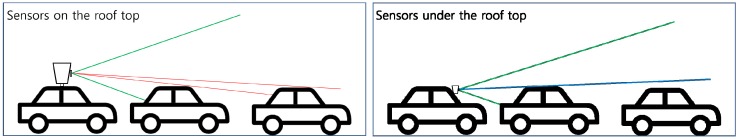
Object detection range depending on sensor position.

Figure 9a is a graphical representation of the recall change of the proposal for the entire ground truth according to the variation of the IoU overlap threshold. What is noteworthy in this graph is that the maximum recovery rate of our method is 81.1%. Given that our proposal generation method detects the LIDAR object that first comes in contact with the vehicle, it can be seen that, in an environment where KITTI data is present and the LIDAR sensor is mounted on the roof of the vehicle, as shown in Figure 10, the proposal is not generated and the recall rate for the entire ground truth is reduced. However, the main reason for detecting objects in a driving environment is to obtain accurate information about nearby objects for safe driving. That is, it is most important to quickly and accurately classify primary collision-capable objects around the car. In addition, the LIDAR sensor is becoming lighter and smaller, making it more likely that the sensor will be located below the roof height when installed in a commercial vehicle. Therefore, when the ground truth object is adjusted to an environment where the LIDAR sensor is installed under the roof of the actual vehicle, the accuracy is much higher than that of the existing MV3D research. Figure 9b shows the recall rate for the IoU overlap threshold change in the adjusted test environment. Ours-E showed a recall rate of 79.3% even when the IoU threshold was 0.7. Figure 9c also shows the recall rate versus the number of proposals when the IoU threshold is 0.25, Figure 9d is 0.5, and Figure 9e is 0.7. Ours-B recalled 97.2%, 92.5% and 71.4%, respectively, in the 41.8 proposals on average, and ours-E recalled 99.3%, 95.5% and 79.3%. These results show that our method can acquire more accurate 3D poses than previous studies when gathering information about surrounding objects with the risk of primary collision at the actual vehicle height.

## 6. Conclusions

In this study, we described an effective way to estimate and restore the poses of 3D objects for autonomous navigation. We used a method of extracting a LIDAR shape to create object-based 2D/3D proposals and complemented the information of a LIDAR feature map. Using the proposed fusion classifier, we confirmed that complementing the object shape of LIDAR feature maps makes object classification more effective. A fusion classifier using two views and three feature sets classifies objects using few proposals generated by the edge orientation of the shape set. Compared with the existing studies, both detection and positional accuracy show better results. Furthermore, it is possible to accurately estimate the *z*-axis position, which is a weak point of the existing research. Also, in this process, the occlusion status between objects can be checked without a separate process. Through this research, we proposed a method to acquire information about objects near the vehicle more quickly and accurately. In the future, we will complement the LIDAR shape set generation method and devise a method to generate the shape of the background object hidden by the foreground object so that it will cover the entire visible area.

## Figures and Tables

**Table 1:** Results from the KITTI database of this study are compared with those obtained using state-of-the-art methods.

		Car			Pedestrian			Cyclist		
Method	Data	E	M	H	E	M	H	E	M	H
Faster R-CNN	Mono	86.71	81.84	71.12	78.51	65.81	61.02	71.56	61.52	55.44
Mono3D	Mono	92.33	88.66	78.96	77.39	66.66	63.44	75.22	63.95	58.96
3DOP	Stereo	93.04	88.64	79.10	82.36	67.46	64.71	80.17	68.81	61.36
VeloFCN	LIDAR	71.06	53.59	46.92						
MV3D	LIDAR + Mono	89.11	87.67	79.54						
ours	LIDAR + Mono	94.91	88.56	80.45	82.70	72.09	60.91	79.43	75.43	61.88

**Table 2:** This table compares the classification accuracy when classifying objects by CCD image alone and concatenating CCD and LiDAR in series. We also compare the classification accuracy of the LiDAR feature with the addition of the LIDAR shape set S. In the ’car’ category, which is a fixed rigid body model of shape, the effect is noticeable.

		Car			Pedestrian			Cyclist		
Method	feature type	E	M	H	E	M	H	E	M	H
CCD only	*C*	89.77	84.15	74.99	78.71	73.01	61.04	74.97	71.90	62.98
Basic Fusion	C+L	89.47	85.52	75.11	79.70	72.01	60.01	78.97	74.90	60.98
LiDAR Extended	C+L+S	94.91	88.56	80.45	82.70	72.09	60.91	79.43	75.43	61.88

**Table 3:** Comparison of IoU rate(%) by difficulty level in KITTI CAR category. We compared the overlap accuracy of the 3D bounding box generated by our method with MV3D, the baseline method. Ours-B represents the basic method of proposal generation and Ours-E represents the extension method.

			Car	
Method	feature type	Easy	Moderate	Hard
MV3D	C+L	59.77	50.15	35.99
Ours-B	*L*	57.81	52.54	32.52
Ours-B	L+S	65.75	60.11	50.87
Ours-E	*L*	70.43	64.58	54.99
Ours-E	L+S	72.91	68.24	64.22
